# Two-step seismic noise reduction caused by COVID-19 induced reduction in social activity in metropolitan Tokyo, Japan

**DOI:** 10.1186/s40623-020-01298-9

**Published:** 2020-11-04

**Authors:** Suguru Yabe, Kazutoshi Imanishi, Kiwamu Nishida

**Affiliations:** 1 Geological Survey of Japan, National Institute of Advanced Industrial Science and Technology (AIST), Tsukuba Central 7, 1-1-1 Higashi, Tsukuba, Ibaraki 305-8567 Japan; 2 Earthquake Research Institute, The University of Tokyo, 1-1-1 Yayoi, Bunkyo-ku, Tokyo, 113-0032 Japan

**Keywords:** Seismic noise, Cultural noise, COVID-19, Tokyo

## Abstract

The COVID-19 pandemic that started at the end of 2019 forced populations around the world to reduce social and economic activities; it is believed that this can prevent the spread of the disease. In this paper, we report an analysis of the seismic noise during such an induced social activity reduction in the Tokyo metropolitan area, Japan. Using seismic data obtained from 18 stations in the Metropolitan Seismic Observation Network (MeSO-net), a two-step seismic noise reduction was observed during the timeline of COVID-19 in Tokyo. The first noise reduction occurred at the beginning of March 2020 in the frequency band of 20–40 Hz. This corresponded with the request by the Prime Minister of Japan for a nationwide shutdown of schools. Although social activity was not reduced significantly at this juncture, local reduction of seismic wave excitation in the high-frequency band, 20–40 Hz, was recorded at some MeSO-net stations located in school properties. The second reduction of seismic noise occurred at the end of March to the beginning of April 2020 in a wider frequency band including lower frequency bands of 1–20 Hz. This timing corresponds to when the Governors of the Tokyo metropolitan area requested citizens to stay home and when the state of emergency was declared for the Tokyo metropolitan area by the government, respectively. Since then, the estimated population at train stations abruptly dropped, which suggests that social activity was severely reduced. Such large-scale changes in social activity affect the seismic noise level in low-frequency bands. The seismic noise level started to increase from the middle of May correlating with increase in population at the train stations. This suggests that social activity restarted even before the state of emergency was lifted at the end of May. The two-step seismic noise reduction observed in this study has not been reported in other cities around the world. Unexpected reduction of social activity due to COVID-19 provided a rare opportunity to investigate the characteristics of seismic noise caused by human activities.
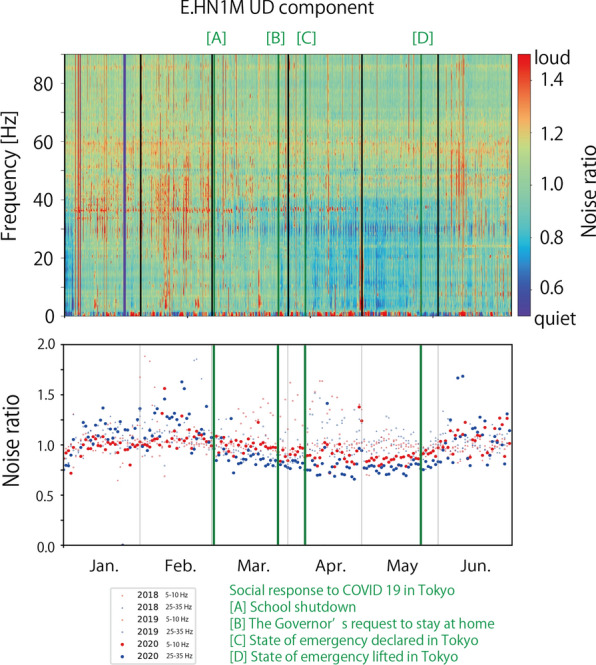

## Introduction

Seismic stations record seismic signals from many sources. This includes seismic noise generated by faint earth tremors because of interactions between the solid earth and meteorological and oceanic phenomena, called a microseism, in the frequency band of 0.05–1 Hz (e.g., Longuet-Higgins 1950; Hasselmann 1963). Conversely, seismic noise in a frequency band higher than 1 Hz is known to show diurnal variability, where a high noise level is recorded during the day (e.g., Bonnefoy-Claudet et al. 2006). This is called cultural noise or anthropogenic noise, as it is known to be excited by human activities such as transportation and machine vibrations (e.g., Gutenberg 1958; Asten 1978; Asten and Henstridge 1984).

The COVID-19 pandemic began at the end of 2019 in China and spread throughout the world (Andersen et al. 2020). To prevent the spread of the disease, reduction in social activity was practiced (e.g., Tian et al. 2020). Earlier observations have indicated that the seismic noise level decreases during long holidays such as Christmas and New Year (Okada and Obara 2000). Seismic noise-level reduction corresponding to social activity reduction for COVID-19 management has been reported around the world (Lecocq et al. 2020; Poli et al. 2020; Xiao et al. 2020; Lindsey et al. 2020). Reduction in social activity is rare, and hence, its occurrence owing to COVID-19 enabled seismologists to understand the characteristics of cultural noise. Seismic noise reduction recorded by seismometers were correlated with mobility data of people in the corresponding cities (Lecocq et al. 2020; Poli et al. 2020; Xiao et al. 2020), suggesting that transportation is the principal noise source. Seismic monitoring with distributed acoustic sensing revealed more detailed spatial changes in social activities in Palo Alto City, California, USA (Lindsey et al. 2020). The study showed that the amount of traffic noise reduction in the city depends on the characteristics of the districts. A district near a grocery store showed approximately 50% reduction in noise, whereas a district near a hospital showed minimal reduction. The amount of seismic noise reduction varies among the cities according to variations in the COVID-19 situation and the corresponding social response (Lecocq et al. 2020; Xiao et al. 2020). Hence, it is important to accumulate observations in various cities to understand the characteristics of cultural seismic noise in urban environments.

**Fig. 1:**
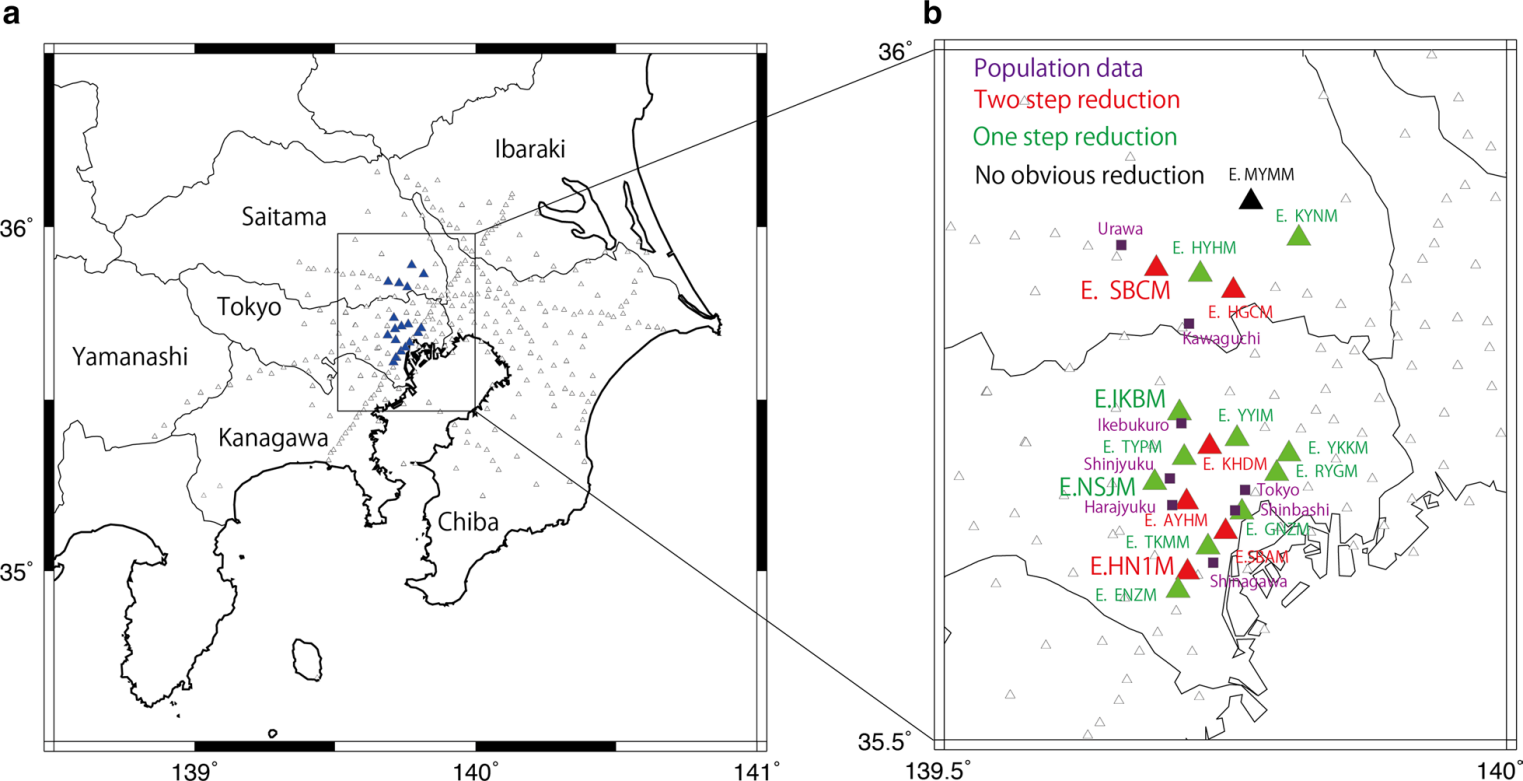
Location map of stations in the MeSO-net in the Tokyo metropolitan area. **a** Map of Kanto region of Japan. Triangles show MeSO-net stations. Blue triangles are the MeSO-net stations considered in this study. **b** Close-up map in the middle of the Tokyo metropolitan area. Red, green, and black triangles show the type of noise reduction observed in this study. Purple squares are train stations for which population data are presented in Fig. 3

This study reports seismic noise reduction caused by social activity reduction for COVID-19 management in the Tokyo metropolitan area. The Metropolitan Seismic Observation Network (MeSO-net), which consists of more than 300 seismic stations, functions in Tokyo and the surrounding prefectures (Sakai and Hirata 2009) (Fig. 1). MeSO-net stations are settled at the bottom of shallow (10–20 m) boreholes and usually located in schools or public park properties. They are close to the ground surface and, thus, more susceptible to seismic waves caused by human activities (Kasahara et al. 2009) than the High-Sensitivity Seismograph Network (Hi-net) situated at a depth of 100 m or more (Okada et al. 2004; Obara et al. 2005), which are often used for the seismic analysis of small earthquakes. Kawakita and Sakai (2009) reported various cultural noise observed via MeSO-net stations. They showed that the background noise level is higher in the central Tokyo metropolitan area than in suburb area, which is attributed to high levels of social activities. They also analyzed the seismic records in MeSO-net stations using running spectrum analysis and determined a high noise level of approximately 50 Hz. As per the researchers, this noise is excited by vibrations of machines such as motors and inverters, which use a commercial electrical power supply with a frequency of 50 Hz. They also documented cultural noise excited by trains and cars. Train signals were observed between 5:00 and 24:00 h, with the busiest period being from 7:00 to 9:00 h, corresponding to the work-commuting time. Strong car signals were observed at MeSO-net stations close to elevated highways. The amount of such cultural noise was expected to decrease because of the social activity reduction during COVID-19 management.

We monitored the temporal changes in the seismic noise level in MeSO-net stations and compared the changes with the timeline of COVID-19 in the Tokyo metropolitan area. We applied polarization analysis (Park et al. 1987) to continuous seismic records of MeSO-net stations to investigate the temporal variations in seismic noise characteristics. We report a two-step seismic noise reduction in different frequency bands correlated with timelines of COVID-19, which was not observed in other cities (Lecocq et al. 2020; Poli et al. 2020; Xiao et al. 2020; Lindsey et al. 2020). We explain the timeline of COVID-19 in Tokyo in the section “Timeline of COVID-19 in the Tokyo metropolitan area”. Seismic data and polarization analyses are explained in the section “Seismic data and methods”. We define the average seismic noise level of each station prior to the COVID-19 pandemic in the section “Average noise level”. Temporal variations of seismic noise relative to the average noise level are discussed in the section “Temporal noise level variations”. The section “Summary” presents a summary.

## Timeline of COVID-19 in the Tokyo metropolitan area

**Fig. 2:**
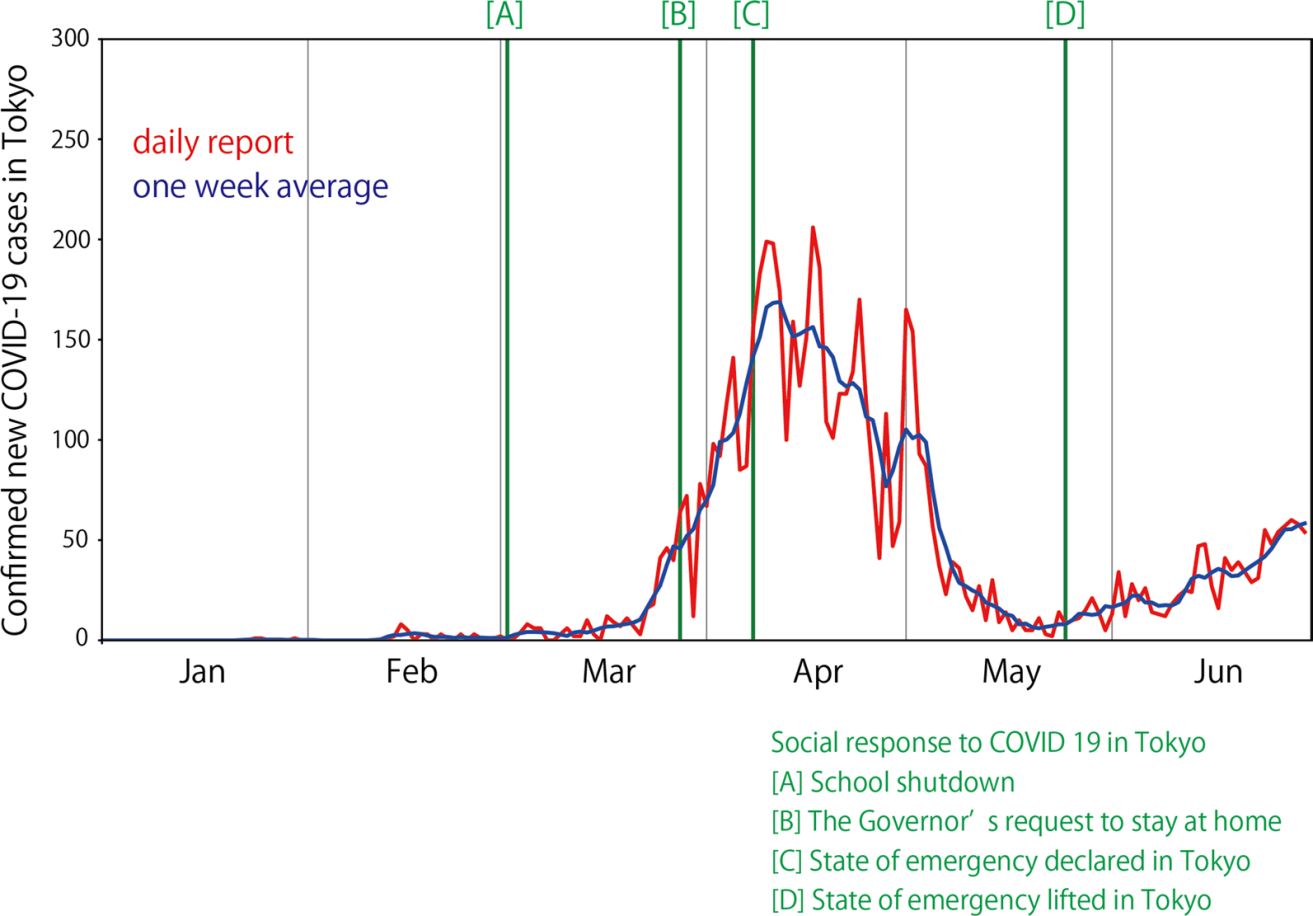
Timeline of COVID-19 in Tokyo. The red curve shows the daily reported number of confirmed new COVID-19 cases in Tokyo. The blue curve is smoothened using a 1-week time window. Green lines indicate the timing of government actions for COVID-19

The COVID-19 disease spread in regions of Japan in January 2020. The first case of COVID-19 in Japan was reported on January 16, 2020 and the first case in Tokyo was reported on January 24, 2020 (Fig. 2). The daily reported number of confirmed new COVID-19 cases was low until February, but started increasing from the beginning of March. The daily reported number exceeded 100 around the beginning of April and it reached its maximum around the middle of April. It started decreasing gradually toward the end of May, though it started increasing again later.

To address this pandemic situation, gradual social activity reduction was requested by the local and national governments. The first step of social activity reduction in Tokyo occurred when the Prime Minister of Japan requested a nationwide shutdown of schools. At this stage, only primary, junior high, and high schools were shut down, though all other economic activities continued. School shutdown began on March 2, 2020 (Fig. 2a) and was enforced until the beginning of June. As the situation worsened, the Governor of the Tokyo metropolitan area requested citizens to stay at home on March 26, 2020 (Fig. 2b). A state of emergency was declared by the national government on April 7, 2020 for the Tokyo metropolitan area (Fig. 2c) and for the entire country on April 16, 2020. We note that the declaration of the state of emergency in Japan did not enforce lockdown of cities and did not legally confine citizens to their homes; citizens were requested to stay indoors voluntarily. As the daily reported number of confirmed new COVID-19 cases decreased, the state of emergency in the Tokyo metropolitan area was lifted on May 25, 2020 (Fig. 2d). Although social distancing is being continuously requested by governments, the daily reported number of confirmed new COVID-19 cases is gradually increasing again after the state of emergency was lifted.

**Fig. 3:**
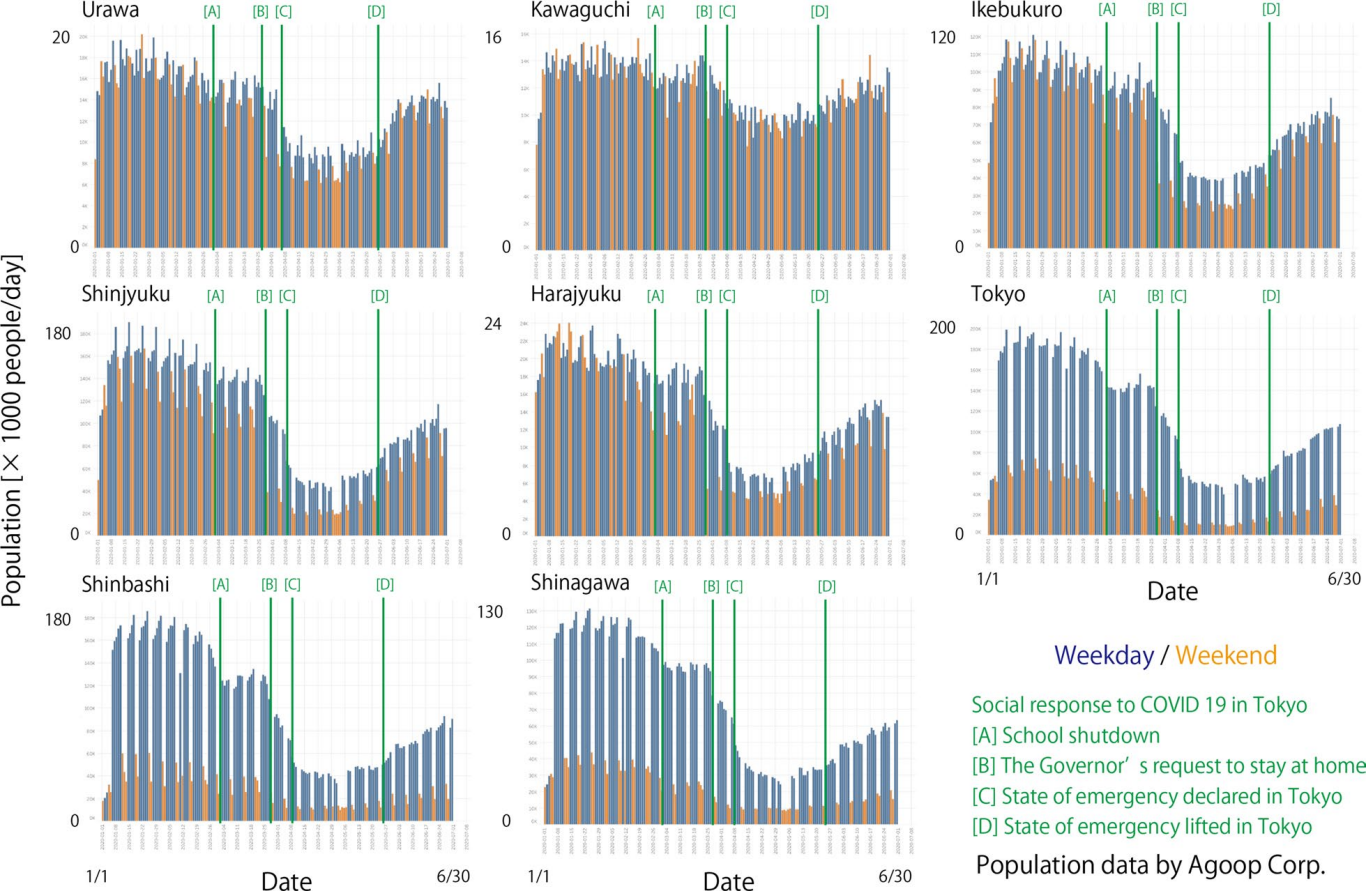
Population at the train stations around MeSO-net stations. Population data were provided by Agoop Corp. This figure is modified from figures originally published by Agoop Corp. on their website (https://corporate-web.agoop.net/pdf/covid-19/agoop_analysis_coronavirus.pdf; accessed on 1 July 2020) with their permission. Blue and orange bars represent daily population around stations for weekdays and weekends, respectively. Green lines represent the timing of government actions for COVID-19

We referred to population data to understand how people reacted to the requests for social activity reduction by governments during the timeline of COVID-19 in Tokyo. Population data were published by Agoop Corp. on their website. The population data were estimated using GPS location information collected from smartphones. Figure 3 shows the estimated populations around train stations near the studied MeSO-net stations. The locations of the train stations are shown in Fig. 1b. At each location, the number of people within a radius of 300–500 m around the train station was counted daily, considering the weight of their staying time within the region.

Population data show that the number of people around the train stations in the Tokyo metropolitan area decreased abruptly at two different times. A small drop occurred when the schools were closed on March 2, 2020. A larger drop occurred from the end of March to the beginning of April when the Governor of the Tokyo metropolitan area requested citizens to stay at home and a state of emergency was declared. By the end of April, the population count decreased to approximately $${\raise0.7ex\hbox{$1$} \!\mathord{\left/ {\vphantom {1 2}}\right.\kern-\nulldelimiterspace} \!\lower0.7ex\hbox{$2$}}$$ to $${\raise0.7ex\hbox{$1$} \!\mathord{\left/ {\vphantom {1 3}}\right.\kern-\nulldelimiterspace} \!\lower0.7ex\hbox{$3$}}$$ of the population in January, which shows that social activity was significantly reduced after the declaration of a state of emergency in the Tokyo metropolitan area. However, this reduction in population did not continue for a long period. After the “Golden Week” in Japan, which is a long holiday period at the beginning of May, the population counts started to increase, even though the state of emergency was not lifted. Although the daily reported number of confirmed new COVID-19 cases kept increasing, the population count around the train stations continued to increase. By the end of June, it recovered to approximately $${\raise0.7ex\hbox{$1$} \!\mathord{\left/ {\vphantom {1 2}}\right.\kern-\nulldelimiterspace} \!\lower0.7ex\hbox{$2$}}$$ to $${\raise0.7ex\hbox{$2$} \!\mathord{\left/ {\vphantom {2 3}}\right.\kern-\nulldelimiterspace} \!\lower0.7ex\hbox{$3$}}$$ of the level in January.

## Seismic data and methods

We studied continuous seismic records of the MeSO-net stations from January 1, 2018 to June 30, 2020. Although MeSO-net consists of more than 300 stations, we selected 18 stations situated in the middle of the Tokyo metropolitan area (Fig. 1). Table 1 shows the MeSO-net stations considered in this study and the types of public property that are present above the seismometers. These MeSO-net stations include servo accelerometers, which have a flat sensitivity to the DC component with a 200-Hz sampling rate. Of the 18 stations presented in Table 1, six stations (E.AYHM, E.KHDM, E.HN1M, E.SBAM, E.SBCM, and E.HGCM) show a two-step seismic noise reduction, whereas the other stations, except for one (E.MYMM), show one-step seismic noise reduction. In this manuscript, we mainly present the results of four stations: two as examples of the two-step reduction (E.HN1M and E.SBCM) and the other two as examples of the one-step reduction (E.IKBM and E.NSJM). The results pertaining to the remaining 14 stations are presented in the Additional file 1.

**Table 1:** List of MeSO-net stations considered in this study

Name	Latitude	Longitude	Ground surface facility
E.HN1M	35.62165	139.71629	Elementary school
E.SBCM	35.84169	139.68864	Junior high school
E.SBAM	35.65107	139.75020	Elementary school
E.AYHM	35.67264	139.71544	High school
E.KHDM	35.71281	139.73606	Elementary school
E.HGCM	35.82589	139.75708	Junior high school
E.IKBM	35.73725	139.70917	Elementary school
E.NSJM	35.68669	139.68708	Elementary school
E.GNZM	35.66521	139.76473	Junior high school
E.HYHM	35.83756	139.72761	High school
E.YYIM	35.71855	139.76035	The University of Tokyo
E.ENZM	35.60844	139.70786	Elementary school
E.TKMM	35.63993	139.73456	Junior high school
E.RYGM	35.69324	139.79560	Elementary school
E.YKKM	35.70734	139.80654	Elementary school
E.TYPM	35.70483	139.71339	Park
E.KYNM	35.86361	139.81531	Park
E.MYMM	35.88994	139.77272	Elementary school

We applied the polarization method (Park et al. 1987) to three-component continuous seismic records. We used 2-s moving time windows with 50% overlap to calculate the Fourier spectra covariance matrix. We considered an hourly average of the complex matrices to ensure stability of the analysis. Then, we calculated the eigen values and eigen vectors of the averaged complex matrices. As the covariance matrix is a Hermitian matrix, all eigen values are real. Azimuth and dip angles for the long axis of polarization were calculated along with the phase differences between two horizontal components and between the horizontal and vertical components from the complex eigen vector for the maximum eigen value.

## Average noise level

**Fig. 4:**
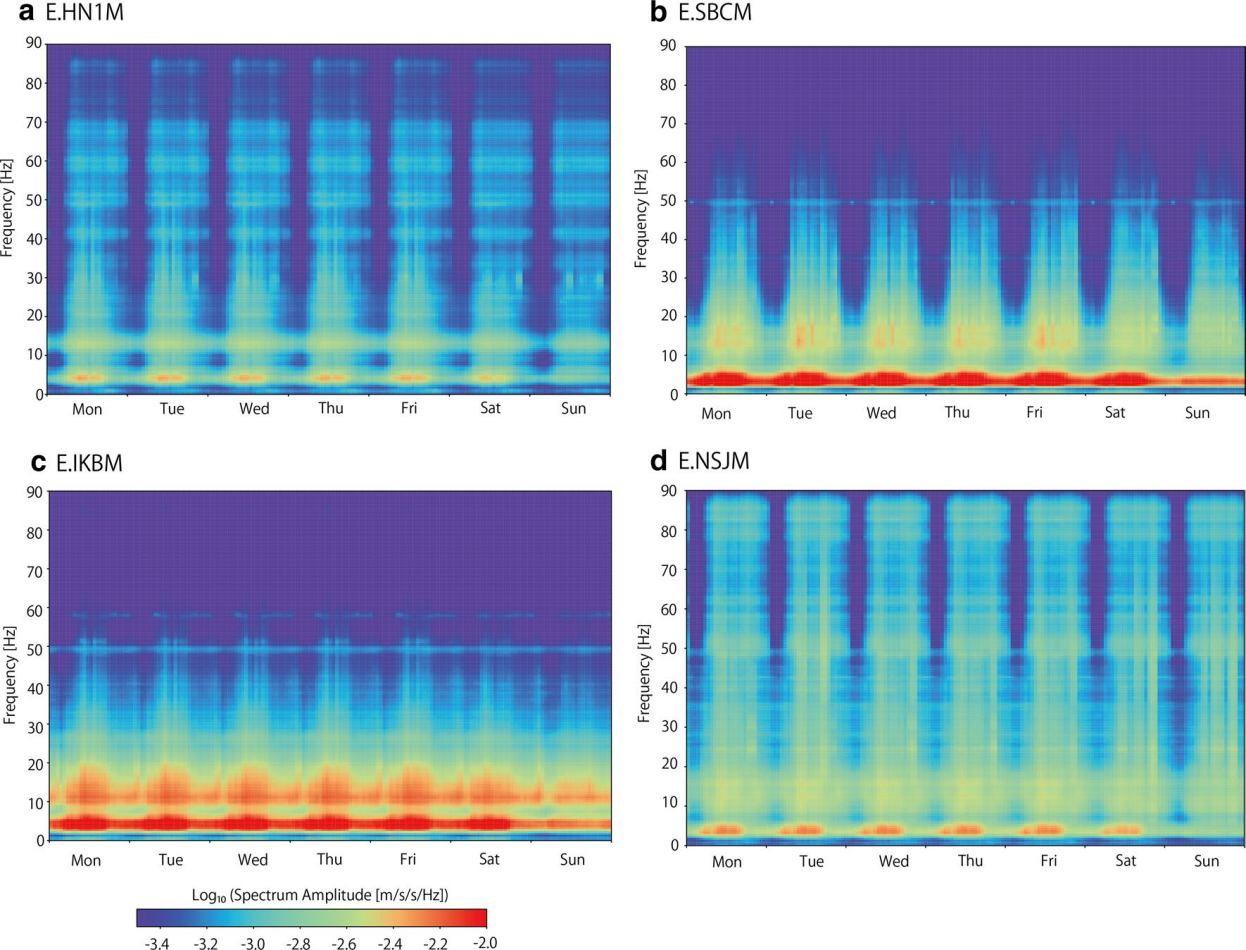
Average noise level of UD components at four stations

To discuss the temporal changes in the seismic noise level, we first defined the average noise level. As the cultural noise-level changes by the day of the week and hour in a day (e.g., Okada and Obara 2000; McNamara and Buland 2004; Marzorati and Bindi 2006; Groos and Ritter 2009), the average noise level needs to be defined every hour and every day of the week. This study uses data from January 1, 2018 to January 31, 2020, when the pandemic was not severe in Japan. The average noise levels were defined for each component by the median value of the square root of the corresponding trace element of the averaged Fourier spectra covariance matrix. Holiday periods, such as national, year-end and new year, and summer holidays, were omitted from the calculation of the average noise level, because the noise level on such days is expected to be lower compared with the usual activity periods. Figure 4 and Additional file 1: Figure S1 show the average noise level of the Up–Down (UD) component during a week.

**Fig. 5:**
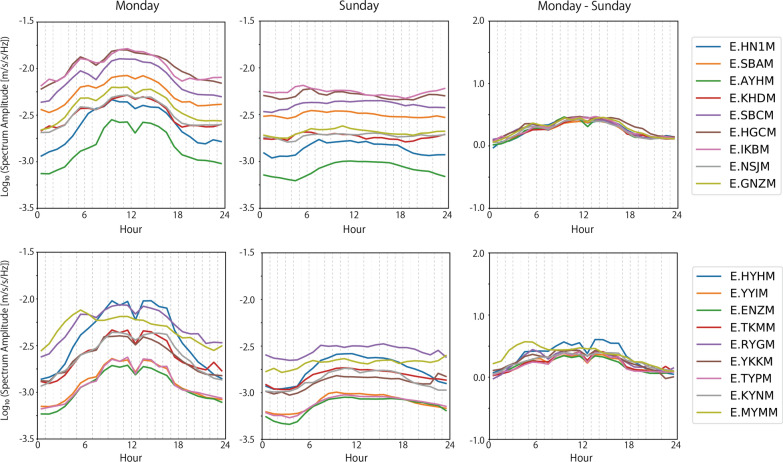
Noise level at 4 Hz on Monday and Sunday. (Left) on Monday. (Middle) on Sunday. (Right) difference between the noise levels on Monday and Sunday

Although the average noise level and its temporal variations vary among the MeSO-net stations, we found several common characteristics. Large noise levels were observed at around 4 Hz throughout a week, although it reduced on a Sunday. Figure 5 compares the noise level of all the studied stations at 4 Hz on Monday and Sunday. On Monday, many stations had a noise peak at 5:00 h. As Kawakita and Sakai (2009) observed, this timing corresponds to when the train service starts. The high noise level also continued between 9:00 and 16:00 h, which corresponds to working hours. A drop in noise level was observed at many stations during lunch break. On Sunday, the daytime noise level was much lower than that of the weekday. Although noise level increased around 5:00 h, prominent noise peaks were not observed. It is interesting to note that the noise-level ratio between Monday and Sunday was almost the same among all the stations. On weekdays, seismic noise was excited, in addition to that observed on Sunday, possibly by economic activity such as heavy transportation and machine vibration from buildings and factories. Such noise sources should be distributed around the city. By considering a ratio of the noise level between weekdays and Sunday, differences in seismic noise amplitude among stations due to site effect are canceled out and differences due to the seismic noise sources are characterized. As the noise-level ratio between weekdays and Sunday has common hourly temporal changes among stations, it is suggested that seismic sources of cultural noise in this frequency band reflect the large-scale social activities in the central Tokyo metropolitan area.

**Fig. 6:**
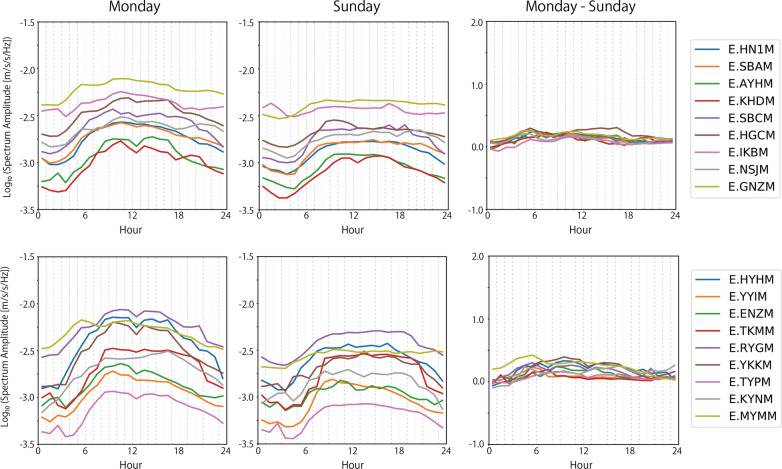
Noise level at 13 Hz on Monday and Sunday. (Left) on Monday. (Middle) on Sunday. (Right) difference between the noise levels on Monday and Sunday

The second prominent characteristic is that large noise signals were also observed at a frequency band of 10–20 Hz. Figure 6 compares the noise level of all the studied stations at 13 Hz on Monday and Sunday. The noise level was also lower on Sunday, as it was in the case of 4 Hz. On Monday, the noise peaks around 5:00 h observed at 4 Hz were less evident in the 10–20 Hz frequency band. High noise level during the day was observed on both Monday and Sunday. The noise level difference between a weekday and Sunday was smaller at 13 Hz than it was in the case of 4 Hz, though its temporal variations were not common among the stations. This suggests that seismic sources of cultural noise excited on weekdays in the 10–20 Hz frequency band reflects more local social activities around the stations.

## Temporal noise-level variations

**Fig. 7:**
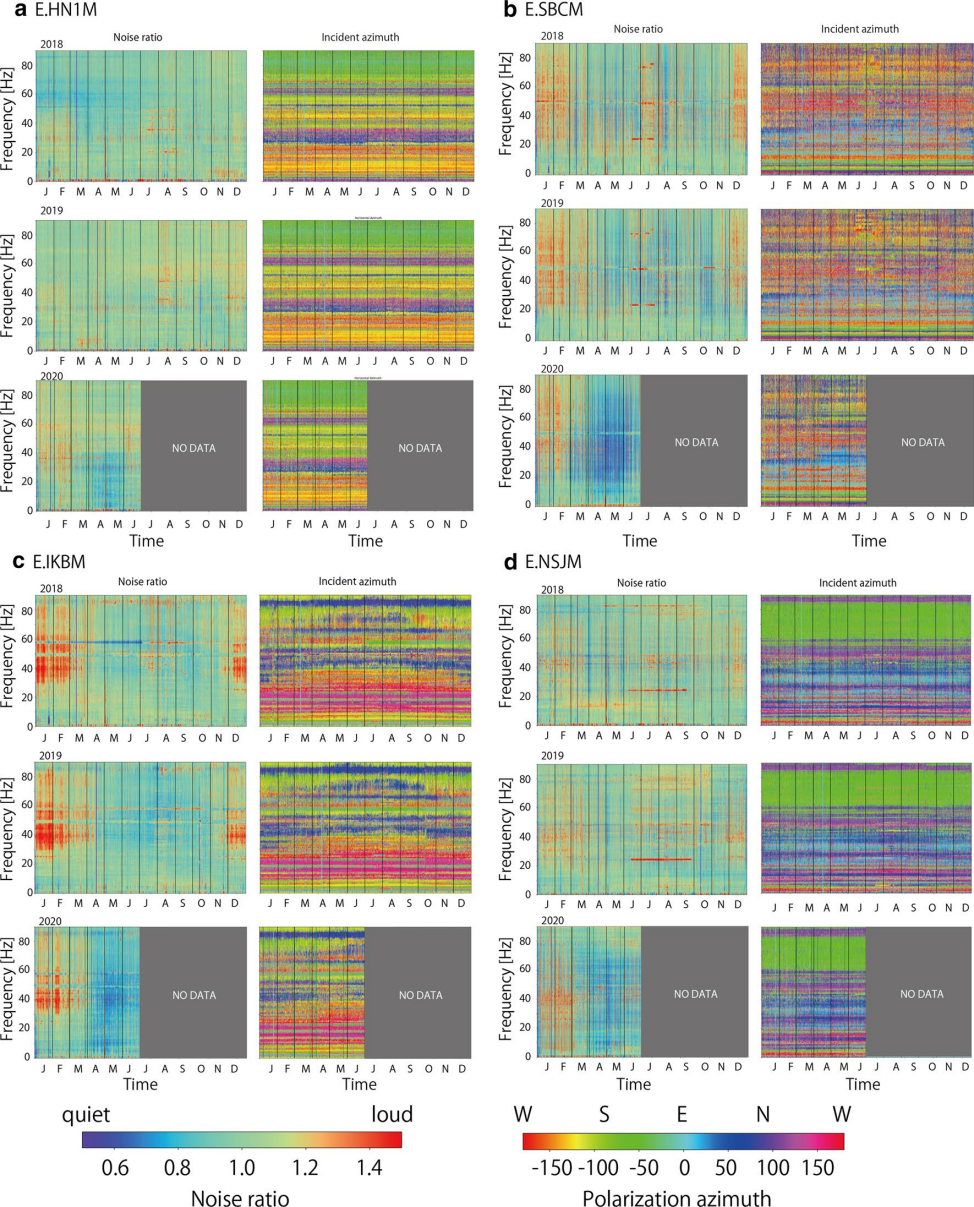
Noise ratio of UD components and polarization azimuth at four stations. The cases of two-step (**a** and** b**) and one-step seismic noise reduction (**c** and** d**)

We monitored temporal variations of seismic noise level in 2.5 years between January 1, 2018 and June 30, 2020. To evaluate temporal variations clearly, we calculated the noise ratio, which is defined as the seismic noise level (square root of the trace element of the averaged Fourier spectra covariance matrix) divided by the average noise level of the corresponding day of the week and hour. Here, holiday periods such as national, year-end and new year, and summer holidays are considered as Sundays. Figure 7 and Additional file 2: Figure S2 show temporal variations in the noise ratio of UD components, as well as the polarization azimuth estimated by polarization analysis.

Temporal variations in the noise ratio in Fig. 7 and Additional file 2: Figure S2 show that the noise level varies throughout the 2.5 years, even before the COVID-19 pandemic influenced social activities. There seem to be seasonal variations in the noise ratio. Winter (December–February) tended to show a higher noise level at many stations, which was especially evident at E.IKBM, E.SBCM (Fig. 7), and E.RYGM (Additional file 2: Figure S2). This high noise level was often observed in the frequency range of > 20 Hz. Summer (around August) also showed a higher noise level at some stations [for example, E.HN1M (Fig. 7), E.SBAM, and E.YKKM (Additional file 2: Figure S2)].

Although noise ratio showed temporal variations, the azimuth and dip angles of noise polarization were constant with time, suggesting that spatial distributions of noise sources were common in the 2.5 years. However, there were several examples where the polarization azimuth changed abruptly or gradually with time. For example, a large noise level was observed from April to October in 2018 and 2019 at E.HGCM station (Additional file 2: Figure S2). Polarization azimuth changed abruptly when the large noise level started and ended. In this case, the polarization azimuth for the low and high noise periods was stable with time, which suggested that spatial distributions of the cultural noise sources around the station changed seasonally.

The noise ratio in 2020 showed a completely different pattern from that in 2018 and 2019 at many stations. The low noise period abruptly started around April at many stations. This low noise level spanned only limited frequency bands such as 1–40 Hz at some stations (for example, E.HN1M), whereas it spanned the entire frequency band at other stations (for example, E.IKBM). Although low noise periods were also observed in 2018 and 2019, the noise level in the low-frequency band of 1–20 Hz was not reduced significantly in 2018 and 2019. As described in the section “Average noise level”, recorded cultural noise in a lower frequency band is suggested to reflect large-scale social activity in the Tokyo metropolitan area (Fig. 5), whereas cultural noise in the higher frequency band is suggested to reflect local social activity around the stations (Fig. 6). Therefore, the seismic noise reduction in the lower frequency band that occurred in 2020 was interpreted to be due to the social activity reduction in the Tokyo metropolitan area owing to COVID-19.

**Fig. 8:**
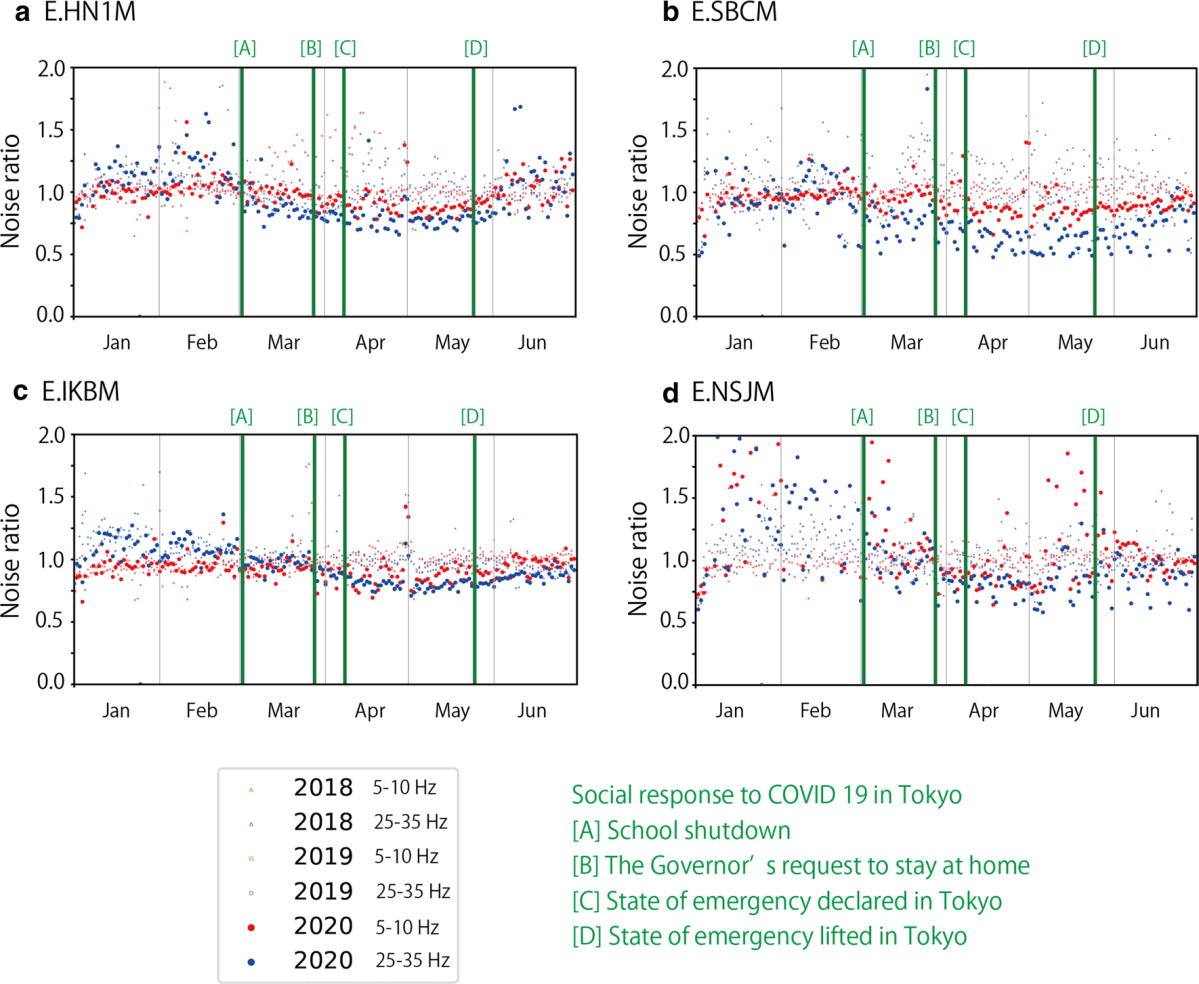
Median noise ratio during the day in two different frequency bands at four stations

At several stations (E.HN1M, E.SBCM, E.AYHM, E.KHDM, E.SBAM, and E.HGCM), seismic noise reduction in a high-frequency band of 20–40 or 20–50 Hz preceded seismic noise reduction observed around April. We calculated the median noise ratio in two different frequency bands (5–10 and 25–35 Hz) during the day (10:00–16:00 h) (Fig. 8 and Additional file 3: Figure S3). The seismic noise reduction in the high-frequency bands occurred when the schools were closed at the beginning of March. As MeSO-net stations are usually installed in school properties, social activity reduction in school buildings decreased seismic wave excitation, which is recorded in the seismometers at the high-frequency bands that are sensitive to locally excited cultural noise. However, this first step of noise reduction was only recorded at a limited number of stations. The relative strength of cultural noise sources around the stations in the higher frequency bands may determine whether seismic noise reduction in the school buildings can be observed.

The seismic noise level started recovering since May 2020. At many stations, the noise ratio increased after the Golden Week, at the beginning of May (Figs. 7, 8, Additional file 2: Figure S2 and Additional file 3: Figure S3). This recovery correlates well with the population recovery (Fig. 3). It started before the state of emergency was lifted on May 25, 2020, which suggests that social activity was restarted before the government officially announced the relaxation.

## Summary

This study reports seismic noise reduction in the Tokyo metropolitan area associated with social activity reduction owing to COVID-19. Although seismic noise reduction associated with the lockdown of cities has already been reported around the world, the MeSO-net stations in the Tokyo metropolitan area show different temporal patterns wherein seismic noise is reduced in two stages correlating with the timeline of COVID-19 in Japan. The first reduction occurred in the frequency band of 20 Hz and higher at the beginning of March when schools were closed. The second reduction occurred in a wider frequency band, including the lower frequency band of 1–20 Hz, from the end of March to the beginning of April when the Governor of the Tokyo metropolitan area requested citizens to stay at home, and when the state of emergency was declared. Many people stopped commuting and social activity was severely reduced during that period. After the middle of May, social activity was gradually restarted, and the seismic noise level increased again.

Two-step noise reduction in different frequency bands is a unique characteristic observed at the MeSO-net stations. When schools were closed at the beginning of March, social activities in the school properties were locally reduced, though social activities in the entire Tokyo metropolitan area were not severely reduced. As many MeSO-net stations are settled in school properties, local reductions of seismic wave excitation were recorded in the high-frequency bands (above 20 Hz). However, noise reduction in this step was observed only in a limited number of stations. If social activities around the stations are high and if the principal noise source in this frequency band is different from the schools, the first step of noise reduction would not be observed. On the other hand, when the state of emergency was declared at the beginning of April, social activities in the entire Tokyo metropolitan area were reduced. Such wide-scale reduction in seismic wave excitation was recorded even in the lower frequency band (1–20 Hz). Irregular cultural noise reduction in the Tokyo metropolitan area due to social activity reduction for COVID-19 showed that the spatial extent of the social activities that affects seismometers is wider for lower frequency noise.

## Supplementary information

**Additional file 1: Figure S1.** Average seismic noise level at the MeSO-net stations other than the four stations shown in Fig. 4.

**Additional file 2: Figure S2.** Noise ratio of UD components and polarization azimuth at the MeSO-net stations other than the four stations shown in Fig. 7.

**Additional file 3: Figure S3.** Median noise ratio during the day in two different frequency bands at the MeSO-net stations other than the four stations shown in Fig. 8.

## Data Availability

MeSO-net data are available at the Hi-net website (https://www.hinet.bosai.go.jp/?LANG=en). Data for confirmed COVID-19 cases in Tokyo were obtained from the website of the Tokyo Metropolitan Government (https://stopcovid19.metro.tokyo.lg.jp/cards/number-of-confirmed-cases/). Population data are published by Agoop Corp. on their website (https://corporate-web.agoop.net/pdf/covid-19/agoop_analysis_coronavirus.pdf).

## Abbreviations

COVID-19: Coronavirus disease of 2019.
Hi-net: High-Sensitivity Seismograph Network
MeSO-net: Metropolitan Seismic Observation Network
UD: Up–Down
