# Supplementary figures and images for: Two-step seismic noise reduction caused by COVID-19 induced reduction in social activity in metropolitan Tokyo, Japan
Source: Earth Planets Space. 2020 Nov 4;72(1):167. doi: 10.1186/s40623-020-01298-9 (PMC7609838; doi:10.1186/s40623-020-01298-9)

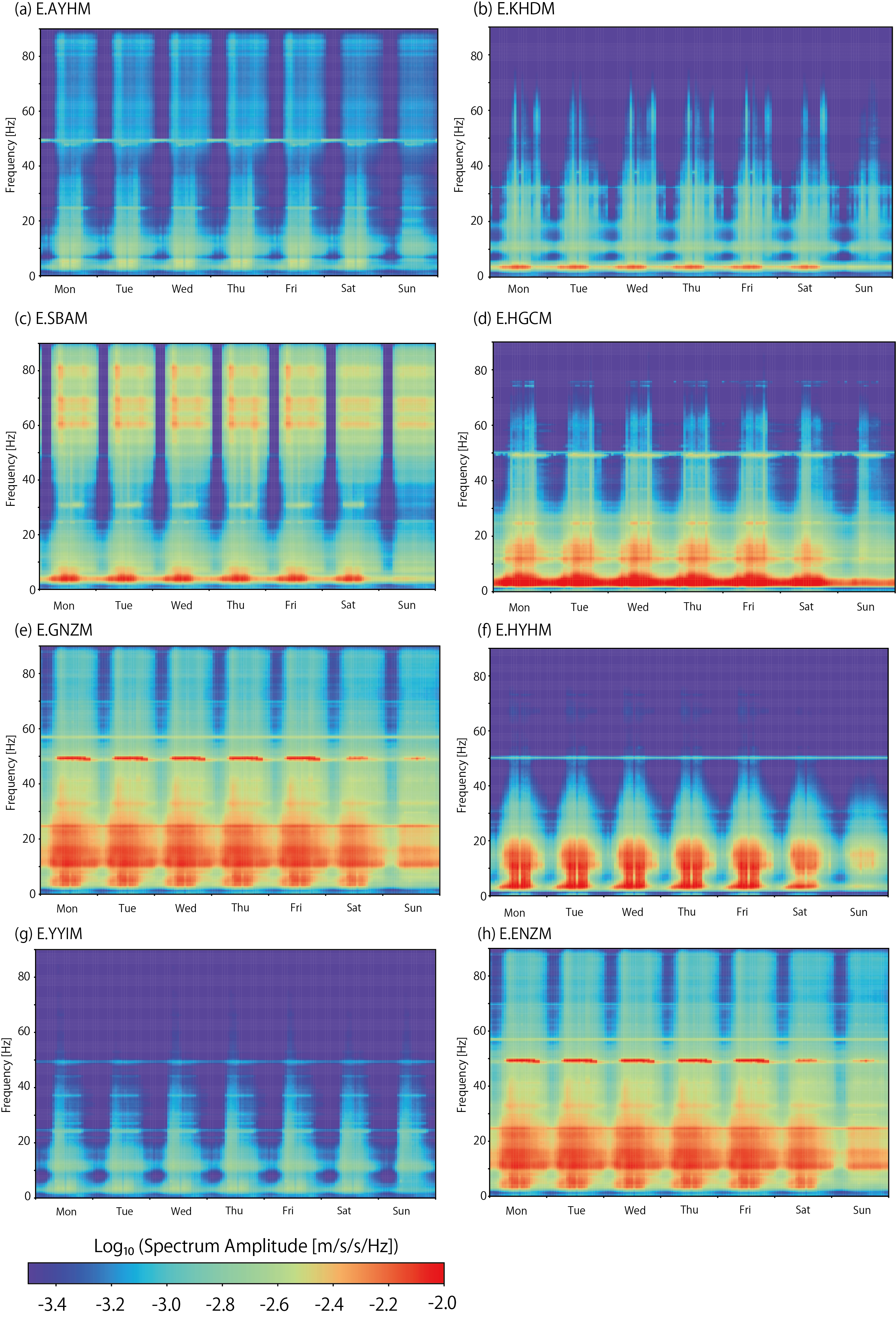


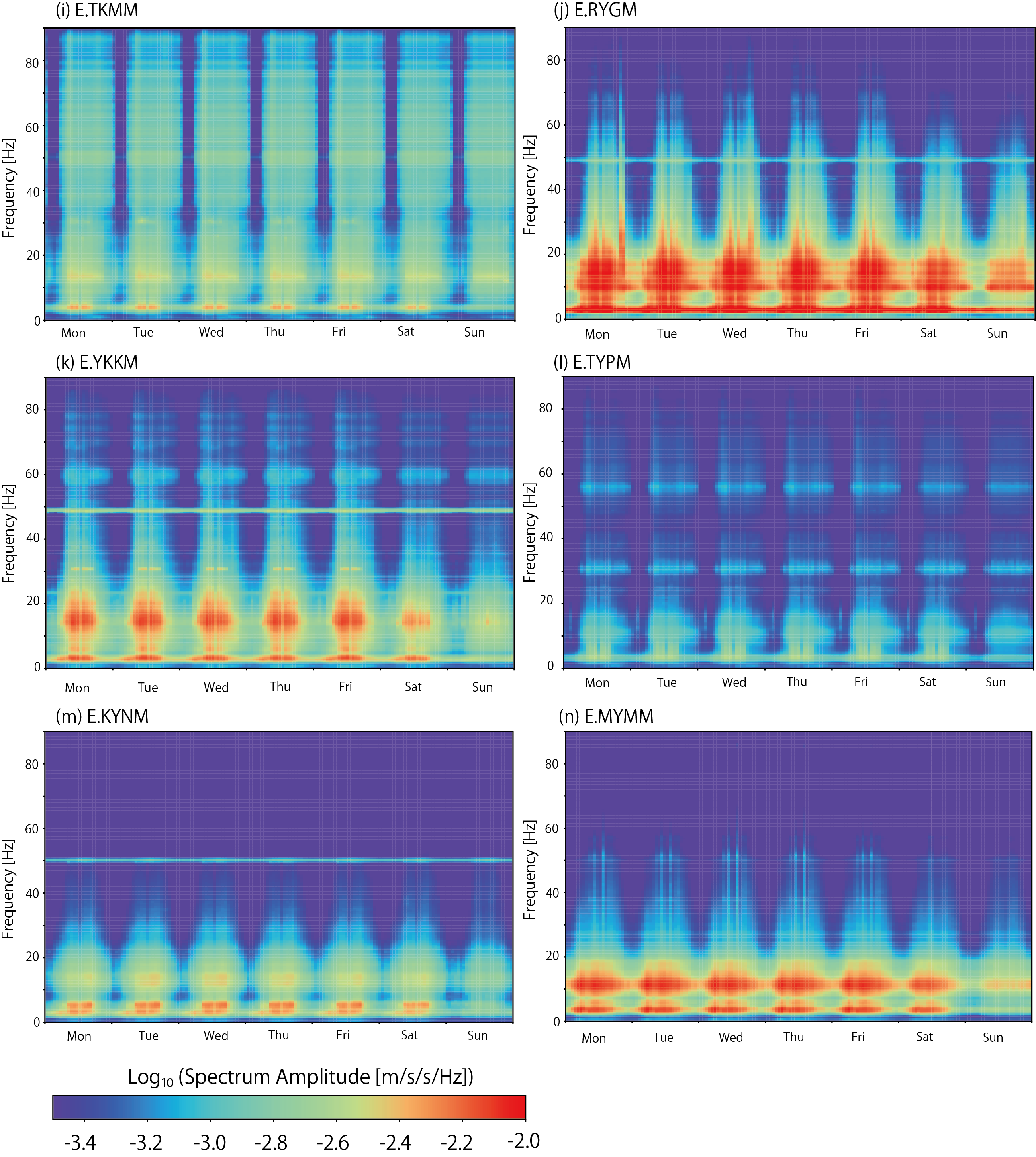

Supplement: Supplementary file 1 — Additional file 1: Figure S1. Average seismic noise level at the MeSO-net stations other than the four stations shown in Fig. 4. [file 40623_2020_1298_MOESM1_ESM.docx]

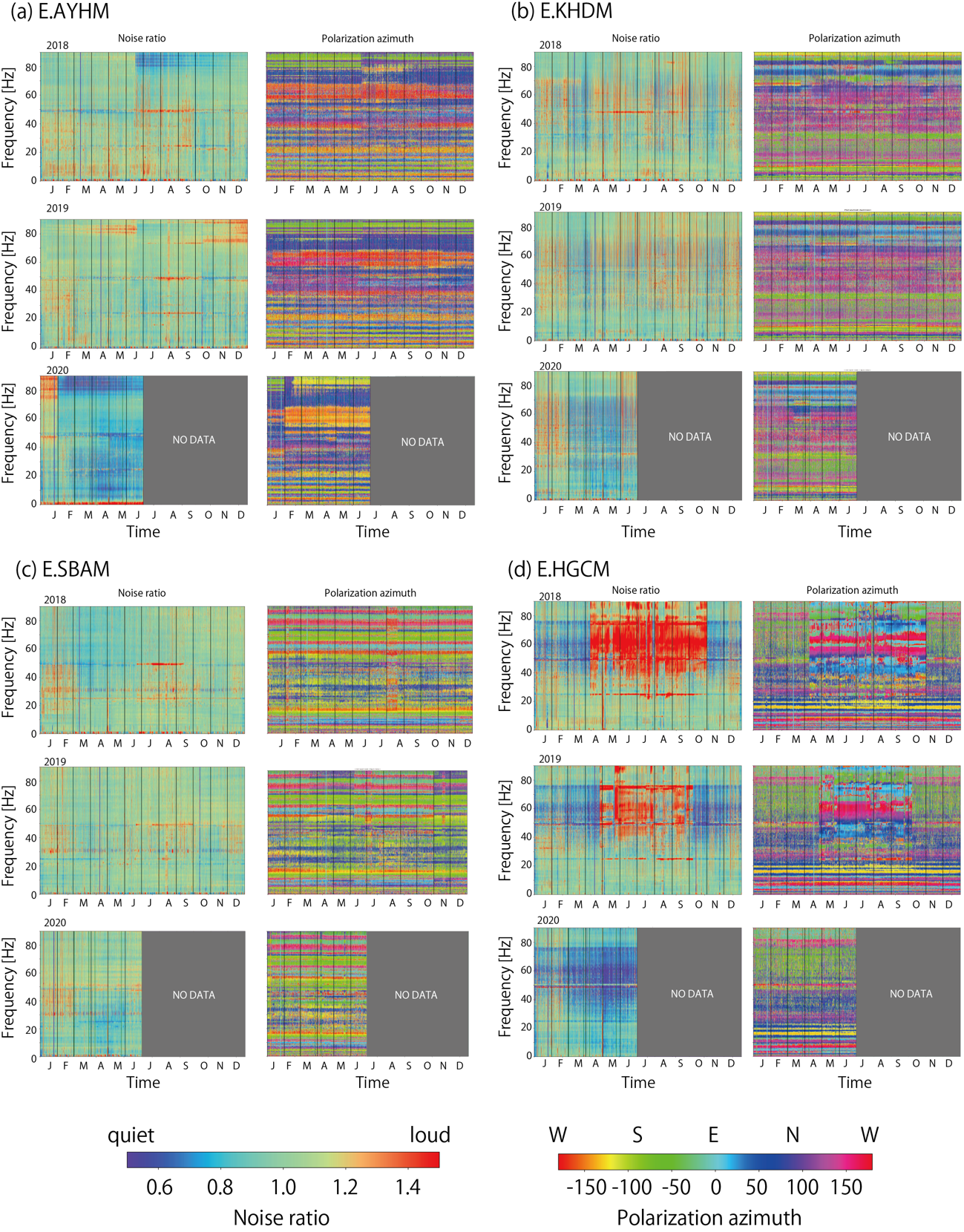


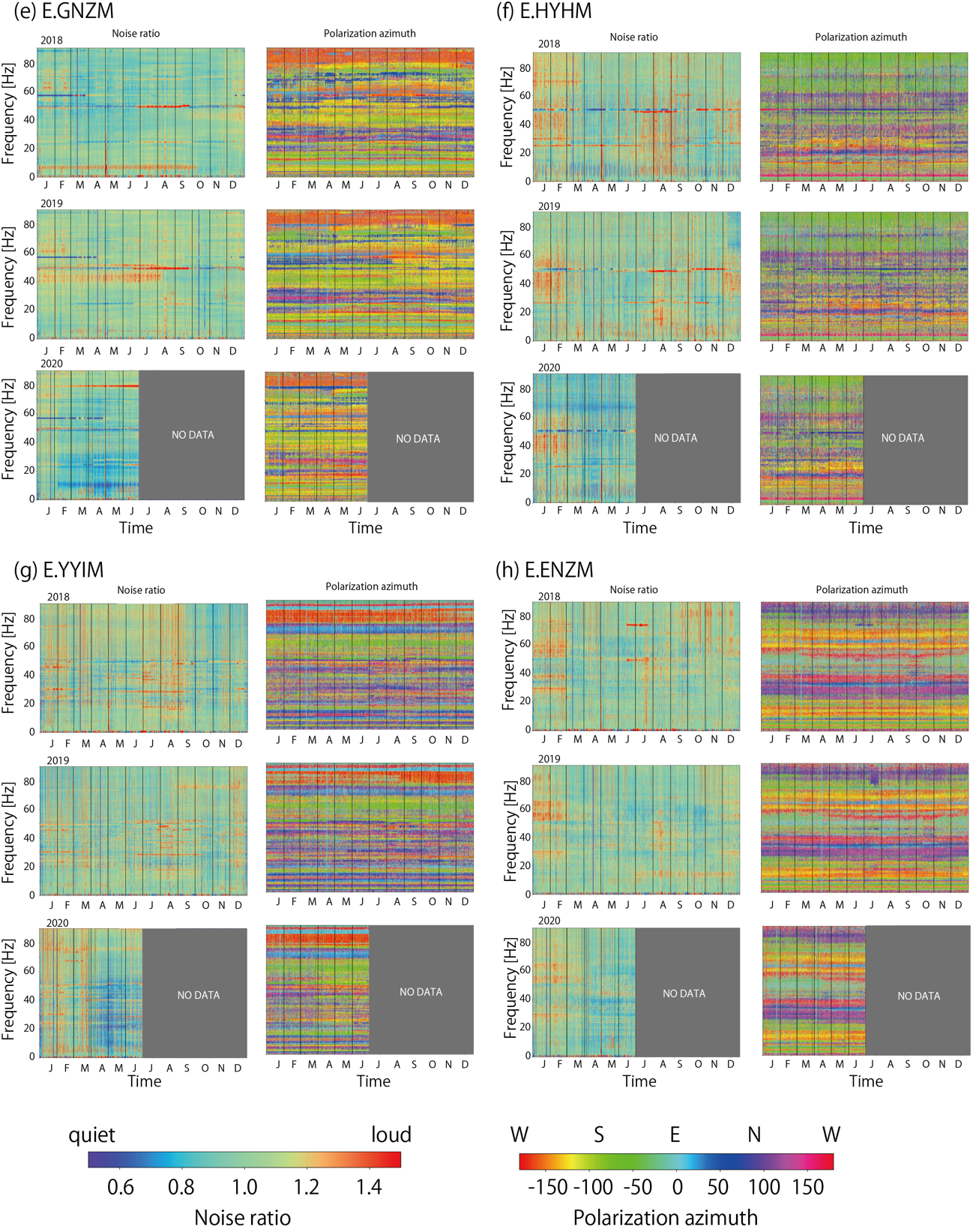


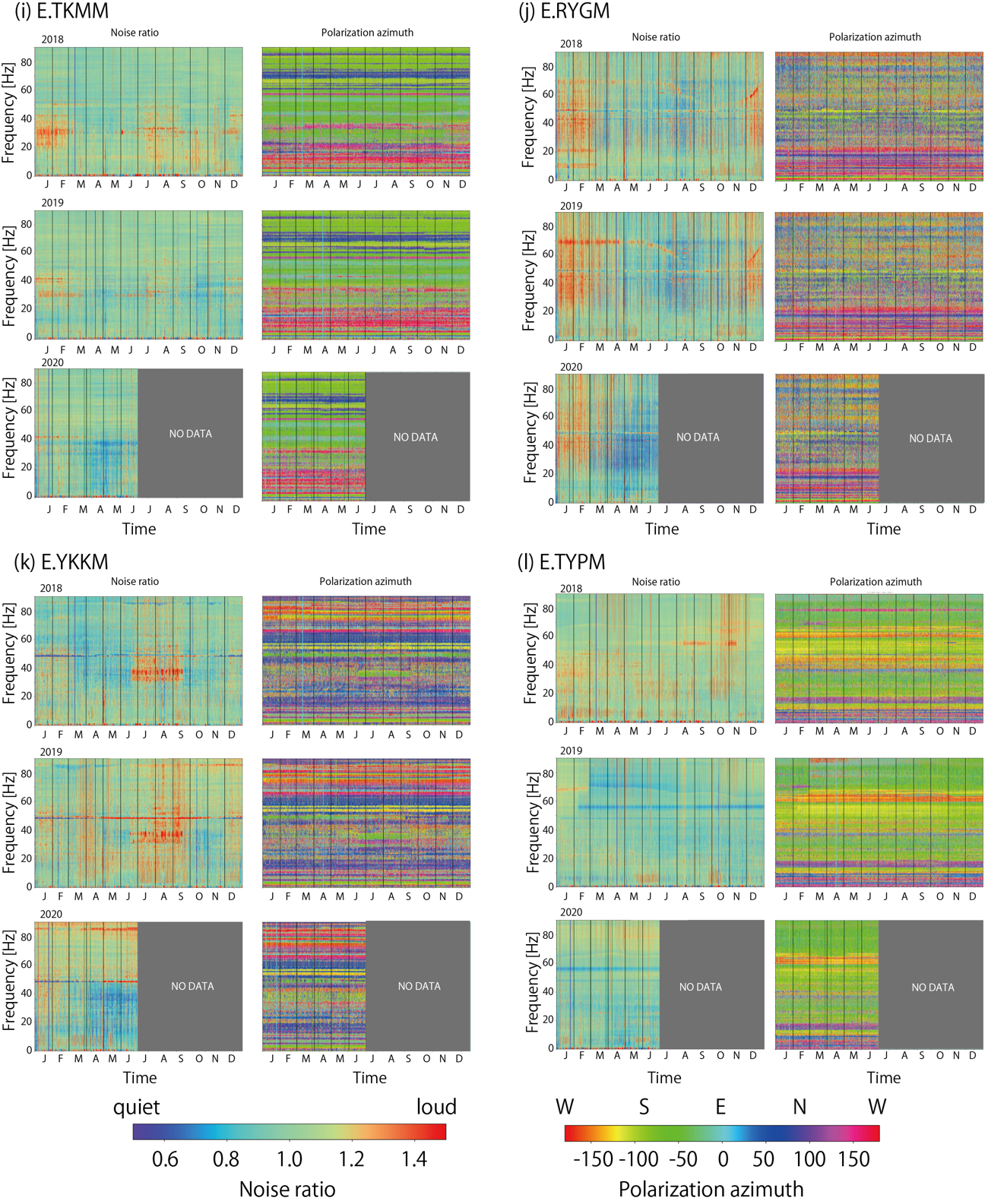


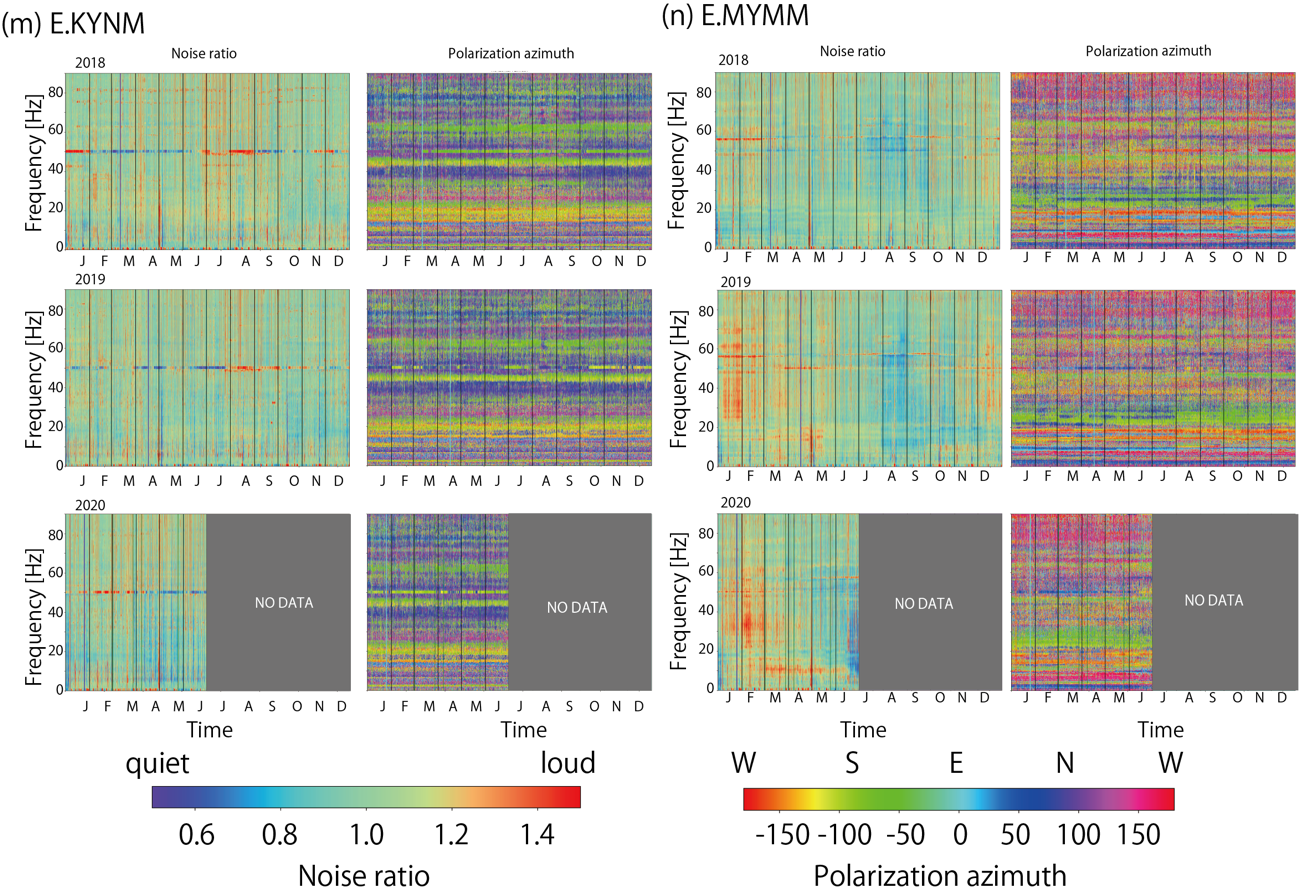

Supplement: Supplementary file 2 — Additional file 2: Figure S2. Noise ratio of UD components and polarization azimuth at the MeSO-net stations other than the four stations shown in Fig. 7. [file 40623_2020_1298_MOESM2_ESM.docx]

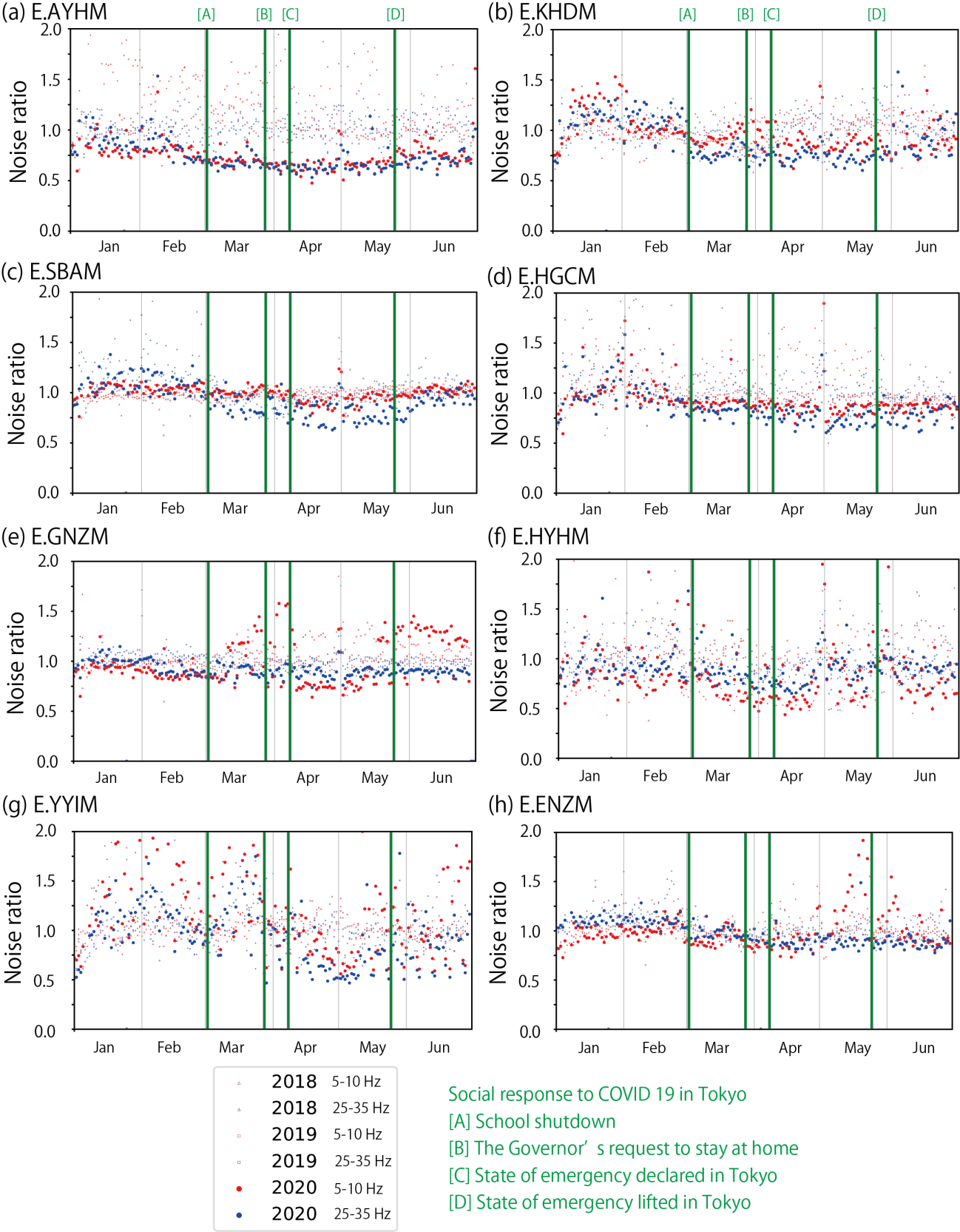

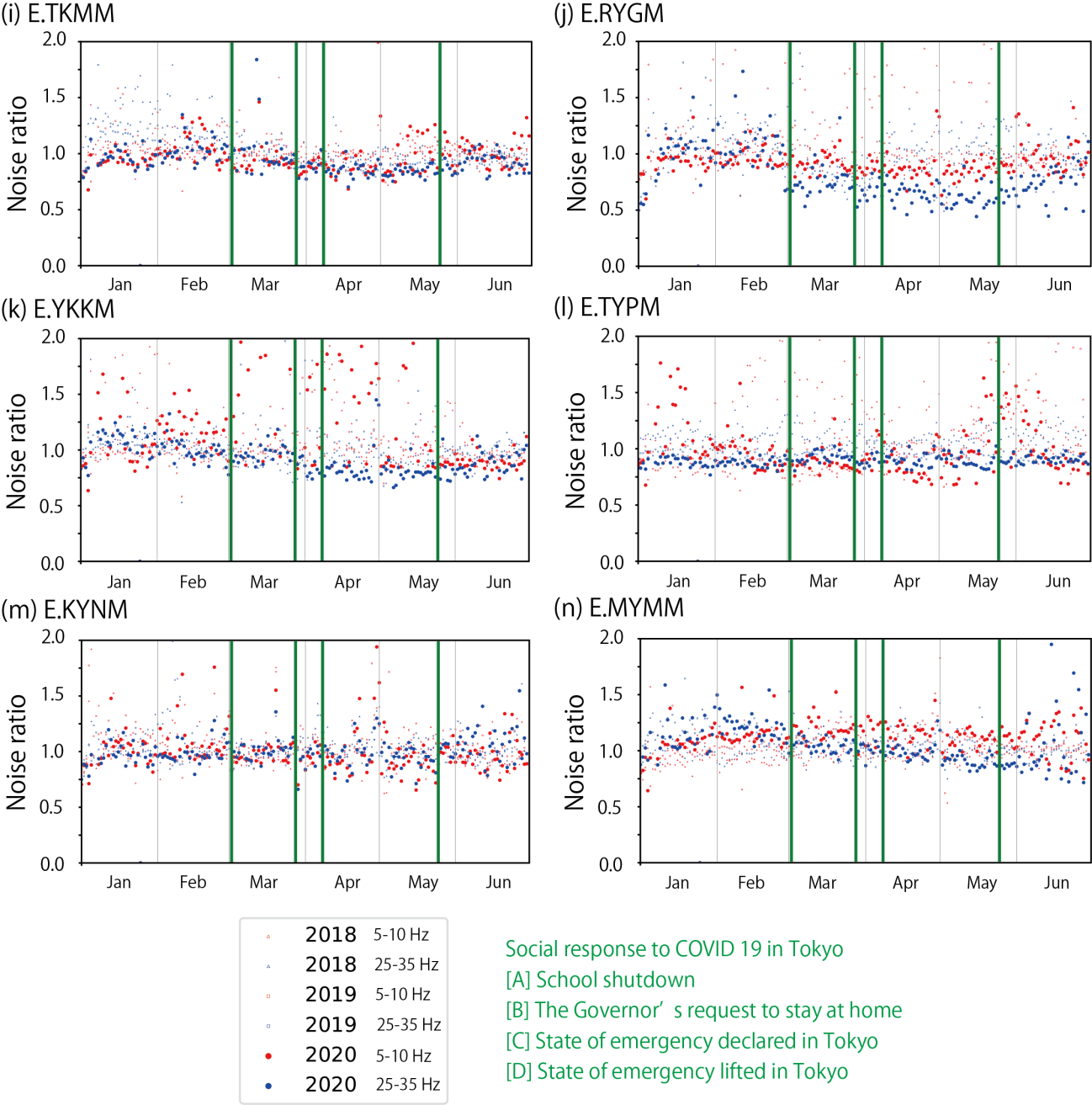

Supplement: Supplementary file 3 — Additional file 3: Figure S3. Median noise ratio during the day in two different frequency bands at the MeSO-net stations other than the four stations shown in Fig. 8. [file 40623_2020_1298_MOESM3_ESM.docx]
